# A Plea for Tube Teeth

**Published:** 1889-02

**Authors:** C. H. Wells

**Affiliations:** Huntingdon, Que.


ARTICLE VII.

### A PLEA FOR TUBE TEETH.

BY C. H. WELLS, L. D. S., HUNTINGDON, QUE.

During a brief practice in the old country some years ago, I was forcibly struck with the many advantages of the English tube over the pin tooth, and the conviction remains with me that even critics here, who have never used them and who therefore are apt to despise them, would probably change their opinion, as I did, could they see the service they render, and bring them into comparison and competition with their rivals. It is true that for facility of application, the pin teeth are superior, but having said that, I know nothing more to be said in their favor. It is complained that the tube teeth, which are only held on by sulphur, draw from the pin; but what about the American teeth? The pivots or pins of the very best often draw from the teeth. You can easily replace a tube tooth which slips from a pin, or you can rivet it on the top and prevent it from slipping, but you cannot restore the tooth which has lost its pins.

One very great advantage of the tube teeth—the pin being immediately in the centre of the tooth,—is that the strain is directly in the middle; the masticating force comes plump in the centre, and is better distributed. In the pin teeth this strain is uneven, and it is common, even in gold plates, to find the attachment of the lining broken from the plate. How frequently, too, does it occur with vulcanite. The whole strain on our bicuspids and molars, is outwards, is not borne by the lower part of the lining, but by the small metal pins in the tooth. But the metal pivot of stiff gold into which the tube tooth is placed, bears strain better, because it is next to impossible to bring pressure on it at any angle, except the tooth itself first breaks, and not often even then.

Another advantage is that to the tongue tube teeth are nearest to nature, and feel best. With ours, the tongue is constantly in contact with metal linings. Another advantage is that with the exception of the specks of solder holding the pins in the plate, there is no quantity of solder likely to cause contractions in the arch. I was told by old British dentists who used tube teeth thirty years ago, that when the journals were discussing the warping of gold plates in this country, they were rarely troubled, owing, they thought, to the absence of a great quantity of solder. A gold plate with tube teeth always fits well if once made well; but the best gold plate with teeth which have been lined and soldered, may be warped any time it has to be repaired, and may be nearly ruined if a botch should repair it with common solder. No botch can spoil a gold plate with tube teeth, because he cannot adapt a new tooth to perfection.

I admit that for close bites, our teeth are better than tube teeth. They can be used too, better with vulcanite combinations. The cheapening of artificial work, not the improvement of it, has given the boom to the pin tooth, and yet among the latest improvements by several manufacturers, we find a modified form of the old tube teeth, with the holes through the sides, and intended for miserable vulcanite to run into, instead of for solid gold pins.

I am using almost exclusively the English tube tooth —with the interior platinum tube lining, when I can get them, in places where ordinary and even improved methods of pivoting is required. They are as dense and as solid as flint, and I have yet to meet the first fiailure on any such account as that friability characteristic of our teeth. I should like to see you cut one of these teeth in two with a pair of scissors, as you can cut clean our gum blocks! You might as well try to take a bite out of a bit of steel.—*Dominion Dental Journal.*

